# Colorectal cancer and thrombosis

**DOI:** 10.1007/s00384-017-2909-2

**Published:** 2017-11-10

**Authors:** P. A. Rees, H. W. Clouston, S. Duff, C. C. Kirwan

**Affiliations:** 1 The University of Manchester, Oxford Road, Manchester, M13 9PL UK; 2 Department of Academic Surgery, Education and Research Centre, University Hospital of South Manchester NHS Foundation Trust, Wythenshawe Hospital, Southmoor Road, Manchester, M23 9LT UK

**Keywords:** Colorectal cancer, Thrombosis

## Abstract

**Significance:**

Colorectal cancer (CRC), results in a hypercoagulable state which manifests clinically as venous thromboembolism (VTE), often presenting as a deep vein thrombosis (DVT) or pulmonary embolism (PE). The consequences of VTE in CRC can be devastating, resulting in long-term morbidity and are a frequent cause of death, even amongst those who would have otherwise had a favourable cancer prognosis.

The incidence of VTE in all cancers is increasing, whilst the exact incidence of VTE in CRC is likely to be underestimated. All cancer treatments increase the risk of VTE in an already at risk population.

**Critical issues:**

CRC-associated VTE is a challenging entity to manage with recurrences occurring more frequently in cancer patients, despite anticoagulation. Anticoagulation, whether treatment or prophylactic, increases the risk of bleeding, especially in patients with cancer. Although strong evidence underpins the initial management of cancer-associated VTE, there is uncertainty with regard optimum treatment duration. For VTE prevention, extended (28 days), pharmacological thromboprophylaxis post CRC surgery is internationally recommended. Pharmacological thromboprophylaxis is not routinely recommended for nonhospitalised patients receiving chemotherapy.

**Future directions:**

There is growing evidence of a symbiotic relationship between cancer biology and the clotting system. Tissue factor (TF), the initiator of the clotting pathway, promotes cancer via clotting dependent and independent mechanisms. Clotting pathway factors, including TF, may have utility as biomarkers in CRC, for assessment of VTE risk in addition to cancer prognosis. The clotting system may also be a target for potential anti-cancer therapies, either via existing anticoagulants or experimental direct TF inhibitors.

## Introduction

Colorectal cancer (CRC) is the 3rd commonest cancer across Europe after cancer of the breast and prostate [1]. In 2012, there were approximately 447,000 new cases diagnosed and 214,000 deaths secondary to CRC [1]. Worldwide, there were 1.36 million new cases diagnosed in the same year, resulting in almost 700,000 deaths [1].

Malignancy leads to a hypercoagulable state. This frequently results in venous thromboembolism (VTE), often presenting as a deep vein thrombosis (DVT) or pulmonary embolism (PE). The risk is heightened further by all cancer therapies.

VTE in the context of CRC is a challenging clinical entity with a growing incidence. It is associated with significant mortality and morbidity. The treatment of CRC patients with VTE is complicated by high recurrence rates and bleeding resulting from therapeutic anticoagulation. In view of this, prophylactic measures to prevent VTE are now well established in all countries across Europe, particularly in the peri-operative period. However, a one size fits all approach persists, whilst the optimum length of treatment and prophylaxis is uncertain.

There is a growing body of evidence supporting a symbiotic relationship between the clotting system and the biology of cancer. CRC leads to increased activation of the clotting system, whilst certain coagulation proteins, e.g. tissue factor (TF), have upregulated expression on CRC tumours. It is possible that this leads to biologically more aggressive cancers, leading to poorer outcomes.

This short communication will explore some of the key issues of VTE in CRC and highlight potential areas of further research.

## Incidence of VTE in colorectal cancer

Malignancy in general is a well-established risk factor for VTE, which occurs in approximately 4% of all hospitalised cancer patients [2]. Patients with cancers of the gastrointestinal tract, including the colorectum, are at particular risk. The incidence of VTE amongst patients with cancer appears to be increasing over time. A large population-based study assessed the trend of VTE incidence between 1995 and 2003 [2]. The proportion of patients diagnosed with a VTE increased from 3.6% in 1995 to 4.6% in 2003, a relative increase of 28% [2]. Perhaps surprisingly, the rate of PE almost doubled, from 0.8 to 1.5%, over the same period [2]. It is possible that the use of more thrombogenic adjuvant therapies, improved diagnostic surveillance, and a greater utilisation of cross-sectional imaging has led, in part, to this increased rate of diagnosis.

Population data suggests that the 2-year cumulative incidence of VTE amongst all patients with CRC is approximately 3% [3]. However, the greatest risk of developing a cancer-associated VTE is in the first 6 months following diagnosis at 5.0% [3]. This subsequently falls to 1.4 and 0.6% in the second 6 months and second year of follow-up, respectively [3]. It is possible that this change in risk over time is related to treatment intensity, particularly surgery and chemotherapy, during the first few months following diagnosis. This may in part, also reflect asymptomatic VTE diagnosed during staging investigations.

Amongst patients undergoing resectional surgery for CRC, the incidence of VTE at 90 days is approximately 2% [4]. This figure has remained relatively stable over time despite an increase in the use of peri-operative thromboprophylaxis [4]. This is possibly related to improved surveillance and the diagnosis of clinically silent VTE.

The increased incidence of VTE amongst patients with cancer is particularly striking amongst those patients undergoing chemotherapy. In recent years, hospitalised patients undergoing chemotherapy have seen an almost doubling in VTE rate from 3.9 to 5.7% [2]. As chemotherapy is utilised in ever more advanced disease and greater numbers of patients, this trend is likely to continue.

The exact incidence of VTE in patients with CRC is uncertain. Most published literature is based on population data which rely heavily on hospital discharge records. As the management of VTE, particularly DVT, moves into the outpatient setting, VTE events are likely to be missed. With regard to surgery-associated VTE, the majority of studies report 30- or 90-day VTE rates. Risk of VTE remains elevated up to 6 months following surgery for colorectal cancer [5]. VTE events may occur beyond these standard windows of follow-up. Also, studies attempting to define the incidence of VTE in CRC will typically report on clinically overt VTEs. Studies rarely screen for incidental VTE, which have the same rates of recurrence and bleeding complications as symptomatic VTE [6]. Therefore, current international guidelines recommend treating them the same as clinically overt VTEs [7]. In summary, the disease burden of VTE amongst patients with CRC is likely to be underestimated.

## Challenges in the treatment and prevention of colorectal cancer-associated VTE

VTE in the context of CRC is difficult to manage. Thromboembolic events are a common cause of death amongst patients with CRC, even in patients who have a good cancer prognosis. VTE is a significant predictor of death within 1 year of cancer diagnosis [3]. It is a particularly strong predictor in patients with local or regional disease, suggesting that VTE may be associated with biologically more aggressive cancers [3].

Anticoagulation is the mainstay of treatment for VTE. This increases the risk of bleeding, particularly in those patients who have recently undergone surgery. Amongst patients with cancer, major bleeding whilst anticoagulated is more common than patients who are cancer-free (12-month cumulative incidence of major bleeding 12.4 vs. 4.9%) [8]. Despite anticoagulation, recurrent VTE rates are increased in patients with cancer compared to those who are cancer-free (12-month cumulative incidence of recurrent VTE 20.7 vs. 6.8%), possibly due to inadequate anticoagulation e.g. secondary to nausea or drug interactions, but more likely as a result of resistant hypercoagulability [8].

Historically, warfarin was the anticoagulant of choice for the treatment of VTE in patients with cancer. However, a landmark paper, randomising patients to receive either low molecular weight heparin (LMWH) or warfarin, demonstrated the superiority of LMWH in preventing recurrent VTE (hazard ratio 0.48; *p* = 0.002) [9]. There was no difference in the rate of major bleeding [9]. Therefore, international guidelines, e.g. the American Society of Clinical Oncology guidelines, recommend 3 to 6 months’ treatment with LMWH for cancer-associated VTE [7]. There is currently no consensus for treatment beyond 6 months.

Mechanical and pharmacological methods of thromboprophylaxis following surgery for CRC have been adopted almost universally. International guidelines currently recommend an extended (28 day) course of LMWH following CRC surgery [7]. This generic approach is unlikely to be appropriate for all patients. For example, in patients with a high nodal burden, the risk of VTE remains elevated up to 6 months following surgery [5]. It is possible that these patients would benefit from an even further extended course of LMWH. Currently, the routine use of LMWH is not recommended for patients undergoing chemotherapy in the outpatient setting [7].

## The colorectal cancer-thrombosis symbiosis and potential for clinical utility

CRC results in upregulation of the clotting system. Under normal physiological conditions TF, the initiator of the extrinsic clotting pathway is expressed by sub-endothelial cells such as smooth muscle cells and stromal fibroblasts. In CRC, TF is abnormally expressed on tumour cells [10]. TF positivity strongly correlates with clinicopathological factors including TNM stage and the presence of hepatic metastasis [10]. TF expression is also associated with poor prognosis. TF-positive CRC is associated with a 3-year survival of 39%, compared to 88% in TF-negative CRC (*p* < 0.001) [10]. As well as being predictive for CRC outcome, TF may have some utility in predicting recurrent VTE in the context of cancer. Patients with high serum TF levels have a significantly increased risk of recurrent VTE whilst on anticoagulation (19 vs. 6%, *p* < 0.001) [11]. Therefore, TF, and other coagulation proteins, may hold utility as biomarkers in CRC.

The clotting pathway promotes cancer by both clotting dependent and independent mechanisms. Clot formation surrounding metastatic cells in the blood stream may allow them to escape immune surveillance to form distant metastatic deposits. Independent of its clotting function, TF can promote intracellular signalling, acting via protease-activated receptor (PAR)-2, which appears to promote key malignant processes including proliferation, migration, angiogenesis, and metastasis [12]. Downstream from TF, thrombin seems to exert similar influence via PAR-1.

The vital relationship between CRC and thrombosis raises the possibility of the clotting pathway being a therapeutic target. Historical studies have hinted at warfarin and LMWH having a possible anti-cancer effect. The potential use of the direct oral anticoagulants e.g. rivaroxaban (ISRCTN14785273) as well as direct TF inhibitors are currently under investigation for their anti-cancer effects.

## Conclusions

VTE in CRC continues to be a challenging clinical entity for patients and clinicians, and its health burden, in the form of chronic pain and venous ulceration, is likely underestimated. Robust strategies for the prevention of VTE in CRC are essential. In the future, evolving knowledge of the symbiotic relationship between cancer and the coagulation system may lead to improved prognostication and the development of novel anti-cancer strategies.
